# ImmunosuppressiveTherapies Differently Modulate Humoral- and T-Cell-Specific Responses to COVID-19 mRNA Vaccine in Rheumatoid Arthritis Patients

**DOI:** 10.3389/fimmu.2021.740249

**Published:** 2021-09-14

**Authors:** Andrea Picchianti-Diamanti, Alessandra Aiello, Bruno Laganà, Chiara Agrati, Concetta Castilletti, Silvia Meschi, Chiara Farroni, Daniele Lapa, Saeid Najafi Fard, Gilda Cuzzi, Eleonora Cimini, Germana Grassi, Valentina Vanini, Roberta Di Rosa, Simonetta Salemi, Gabriele Nalli, Andrea Salmi, Federica Repele, Anna Maria Gerarda Altera, Gaetano Maffongelli, Claudia Palazzolo, Serena Vita, Sara Leone, Vincenzo Puro, Maria Rosaria Capobianchi, Giuseppe Ippolito, Emanuele Nicastri, Delia Goletti

**Affiliations:** ^1^Department of Clinical and Molecular Medicine, “Sapienza” University, S. Andrea University Hospital, Rome, Italy; ^2^Translational Research Unit, National Institute for Infectious Diseases Lazzaro Spallanzani-IRCCS, Rome, Italy; ^3^Laboratory of Cellular Immunology, National Institute for Infectious Diseases Lazzaro Spallanzani-IRCCS, Rome, Italy; ^4^Laboratory of Virology, National Institute for Infectious Diseases Lazzaro Spallanzani-IRCCS, Rome, Italy; ^5^Unità Operativa Semplice (UOS) Professioni Sanitarie Tecniche, National Institute for Infectious Diseases Lazzaro Spallanzani-IRCCS, Rome, Italy; ^6^Clinical Division of Infectious Diseases, National Institute for Infectious Diseases Lazzaro Spallanzani-IRCCS, Rome, Italy; ^7^UOC Emerging Infections and Centro di Riferimento AIDS (CRAIDS), National Institute for Infectious Diseases Lazzaro Spallanzani-IRCCS, Rome, Italy; ^8^Scientific Direction, National Institute for Infectious Diseases Lazzaro Spallanzani-IRCCS, Rome, Italy

**Keywords:** COVID-19, mRNA vaccine, rheumatoid arthritis, whole blood, T cell response, antibody response, DMARD (disease modifying anti-rheumatic drug), biological therapy

## Abstract

**Objective:**

To assess in rheumatoid arthritis (RA) patients, treated with different immunosuppressive therapies, the induction of SARS-CoV-2-specific immune response after vaccination in terms of anti-region-binding-domain (RBD)-antibody- and T-cell-specific responses against spike, and the vaccine safety in terms of clinical impact on disease activity.

**Methods:**

Health care workers (HCWs) and RA patients, having completed the BNT162b2-mRNA vaccination in the last 2 weeks, were enrolled. Serological response was evaluated by quantifying anti-RBD antibodies, while the cell-mediated response was evaluated by a whole-blood test quantifying the interferon (IFN)-γ-response to spike peptides. FACS analysis was performed to identify the cells responding to spike stimulation. RA disease activity was evaluated by clinical examination through the DAS28crp, and local and/or systemic clinical adverse events were registered. In RA patients, the ongoing therapeutic regimen was modified during the vaccination period according to the American College of Rheumatology indications.

**Results:**

We prospectively enrolled 167 HCWs and 35 RA patients. Anti-RBD-antibodies were detected in almost all patients (34/35, 97%), although the titer was significantly reduced in patients under CTLA-4-inhibitors (median: 465 BAU/mL, IQR: 103-1189, p<0.001) or IL-6-inhibitors (median: 492 BAU/mL, IQR: 161-1007, p<0.001) compared to HCWs (median: 2351 BAU/mL, IQR: 1389-3748). T-cell-specific response scored positive in most of RA patients [24/35, (69%)] with significantly lower IFN-γ levels in patients under biological therapy such as IL-6-inhibitors (median: 33.2 pg/mL, IQR: 6.1-73.9, p<0.001), CTLA-4-inhibitors (median: 10.9 pg/mL, IQR: 3.7-36.7, p<0.001), and TNF-α-inhibitors (median: 89.6 pg/mL, IQR: 17.8-224, p=0.002) compared to HCWs (median: 343 pg/mL, IQR: 188-756). A significant correlation between the anti-RBD-antibody titer and spike-IFN-γ-specific T-cell response was found in RA patients (rho=0.432, p=0.009). IFN-γ T-cell response was mediated by CD4^+^ and CD8^+^ T cells. Finally, no significant increase in disease activity was found in RA patients following vaccination.

**Conclusion:**

This study showed for the first time that antibody-specific and whole-blood spike-specific T-cell responses induced by the COVID-19 mRNA-vaccine were present in the majority of RA patients, who underwent a strategy of temporary suspension of immunosuppressive treatment during vaccine administration. However, the magnitude of specific responses was dependent on the immunosuppressive therapy administered. In RA patients, BNT162b2 vaccine was safe and disease activity remained stable.

## Introduction

The COronaVIrus Disease-2019 (COVID-19) pandemic caused by the Severe Acute Respiratory Syndrome CoronaVirus 2 (SARS-CoV-2) has recently emerged as a new human-to-human transmissible disease with a serious global health impact and still difficult clinical management (1–3).

Mass vaccination is the single most effective public health measure for controlling the COVID-19 pandemic, and a global effort to develop and distribute an effective vaccine produced important containment results. Several data are currently available about efficacy of mRNA platform vaccines, namely BNT162b2 and mRNA-1273 vaccines, in inducing strong antibody and cell-mediated immune responses in naïve healthy individuals (4–6). The ability to elicit a coordinated induction of both humoral- and cell-mediated arms is fundamental for a more effective fighting of SARS-CoV-2 infection (7, 8).

Currently available data suggest that patients with autoimmune inflammatory rheumatic diseases have a slightly higher prevalence of SARS-CoV-2 infections, risk of hospitalization, and death from COVID-19 than the general population, and they have been considered a priority target group for vaccine administration (9, 10). However, considering the immunologic dysregulation and the immunosuppressive treatments frequently adopted in these patients, some concerns have arisen regarding vaccine efficacy and safety.

Recently, some encouraging data on mRNA vaccination in rheumatoid arthritis (RA) patients have emerged from few small and one large prospective observational multicenter study evaluating the immunogenicity and safety of the BNT162b2 mRNA vaccine compared to control subjects without rheumatic diseases (11–13). Overall, these studies show that the antibody response to BNT162b2 vaccine is immunogenic in the majority of patients with RA (86-100%), but delayed and reduced compared to controls. Although the results on the impact of the immunosuppressive therapy on vaccine immunogenicity are not homogenous, most of studies suggest that rituximab followed by abatacept, mycophenolate mofetil, corticosteroids (CCS), and methotrexate (MTX) can induce a significant reduction of seropositive rate and antibody levels (14). These data are crucial to optimize the management of RA patients and to improve vaccine safety and effectiveness, but they need to be confirmed and supplemented by additional real-world studies and by the evaluation of the T-cell-specific response.

This study aimed to assess in RA patients treated with different immunosuppressive therapies the induction of a specific immune response after SARS-CoV-2 vaccination in terms of anti-region-binding-domain (RBD)-antibody- and T-cell-specific responses against spike, and the safety of vaccination in terms of clinical impact on disease activity. A cohort of health care workers (HCWs) was used as a healthy control group.

## Materials and Methods

### Study Population

Participants were enrolled from two parallel prospective studies conducted at the National Institute for Infectious Diseases (INMI) Lazzaro Spallanzani and approved by the INMI Ethical Committee. The approved studies evaluated the immune response to SARS-CoV-2 vaccination in both HCWs enrolled at INMI (approval number 297/2021) and in rheumatologic patients enrolled at Sant’Andrea Hospital in Rome (approval number 318/2021). All HCWs and rheumatologic patients received the BNT162b2-mRNA vaccine. Inclusion criteria for the enrollment of rheumatologic patients were: a diagnosis of RA according to the European League Against Rheumatism/American College of Rheumatology (EULAR/ACR) 2010 criteria (15), having completed the two-dose schedule of the mRNA vaccine in the last 2 weeks, being on treatment with a biological drug (except anti-CD20) with or without MTX or other disease modifying anti-rheumatic drugs (DMARD), with only DMARD, with anti-Janus kinase (JAK) or low dosage of CCS (prednisone <7.5 mg/day or methylprednisolone <6 mg/day). Written, informed consent was required to consecutively enroll patients and controls.

### Study Procedures

Clinical, demographic data and the use of medication were collected at the time of enrollment (T0) and after 2 weeks from the second dose (T1) (**Table 1**). RA disease activity was evaluated by clinical examination at T0 and T1 through the DAS28crp. At T1, blood samples were collected and local and/or systemic clinical adverse events were registered.

**Table 1:** Demographical and clinical characteristics of the 202 enrolled subjects.

Characteristics		RA patients	HCWs	P value
**N (%)**		35 (17.3)	167 (82.7)	
**Age median (IQR)**		59 (55–65)	42 (32–53)	<0.0001*
**Male N (%)**		8 (22.9)	48 (28.7)	0.298^§^
**Origin N (%)**	**West Europe**	31 (88.6)	165 (98.8)	0.0050^§^
	**East Europe**	2 (5.8)	2 (1.2)	
	**Africa**	1 (2.8)	0 (0)	
	**Sud America**	1 (2.8)	0 (0)	
**Rheumatologic treatment N (%)**	**TNF-α-inhibitors +/- DMARD**	7 (20)	–	
	**IL-6-inhibitors+/-DMARD/CCS**	8 (22.9)	–	
	**CTLA-4-inhibitors +/-DMARD/CCS**	13 (37.1)	–	
	**DMARD +/- CCS**	7 (20)	–	
**Disease activity** **median (IQR)**	**DAS28crp T0**	3.2 (2.5-3.5)	–	0.732*
	**DAS28crp T1**	3.2 (2.0-3.5)	–	
**Therapy**	**Years**	4.9 (1.9-8.0)	–	
**Lymphocytes count N (%)**		32 (91.4)	0 (0)	
**Lymphocytes count N (%)** **Median x10^3^/µL (IQR)**	**TNF-α-inhibitors +/- DMARD**	7 (21.9) 1.97 (1.07-4.01)	–	0.067^#^
	**IL-6-inhibitors+/-DMARD/CCS**	7 (21.9) 1.44 (0.74-1.71)	–	
	**CTLA-4-inhibitors +/-DMARD/CCS**	11 (34.3) 2.07 (1.75-2.84)	–	
	**DMARD +/- CCS**	7 (21.9) 1.37 (1.26-1.86)	–	

DMARD, disease modifying antirheumatic drugs; CCS, corticosteroids; RA, rheumatoid arthritis; DAS28, disease activity score 28; N, number; IQR, interquartile range; *Mann-Whitney U-statistic test; ^§^Chi-square test; ^#^Kruskal-Wallis test.

RA patients were stratified according to drug treatments in four groups: TNF-α-inhibitors with or without DMARD, IL-6-inhibitors with or without DMARD/CCS, CTLA-4-inhibitors with or without DMARD/CCS, and DMARD with or without CCS. The lymphocyte count of the RA patients was performed within one week from the samples' collection taken for the immune-based assays.

A convenient sample of 167 individuals was included as healthy controls from the cohort of vaccinated HCWs at INMI L. Spallanzani (4, 16).

### IFN-γ Whole-Blood Assay

Whole-blood (600 µL) was stimulated with a pool of peptides covering the sequence of SARS−CoV−2 spike protein (SARS−CoV−2 PepTivator^®^ Prot_S1, Prot_S, and Prot_S+, Miltenyi Biotec, Germany) in a 48-well flat-bottom plate (17). The PepTivator^®^ Peptide Pools used were constituted by peptides of 15 amino acid length with an 11 amino acid overlap. After 20-24 hours of incubation at 37°C (5% CO_2_), plasma was harvested and stored at -80°C until use. IFN-γ levels were quantified in the plasma samples using an automatic ELISA (ELLA, protein simple). IFN-γ values of the stimulated samples were subtracted from the unstimulated-control value. The detection limit of this assay was 0.17 pg/mL.

### Peripheral Blood Mononuclear Cells (PBMCs) and *In Vitro* Stimulation

PBMCs, isolated from HCWs (n=7) and RA patients (n=15), were thawed, counted, assessed for viability, and rested for 2-4 hours at 37°C in RPMI supplemented with 1% L-glutamine, 1% penicillin/streptomycin (Euroclone S.p.A, Italy), and 10% heat-inactivated FBS. For antigen-specific T-cell stimulation, PBMCs were seeded at a concentration of 2.5 × 10^6^ cells/mL in a final volume of 200 µL in a 96-multiwell flat-bottom plate (COSTAR, Sigma Aldrich), and stimulated with spike peptide pool at 1 µg/mL or Staphylococcal Enterotoxin B (SEB) at 200 ng/mL, used as a positive control. We added anti-CD28 and anti-CD49d monoclonal antibodies (BD Biosciences San Jose, USA) to co-stimulate cells at a final concentration of 1 µg/mL each. After 1h of incubation at 37°C (5% CO_2_), a Golgi plug (BD Biosciences) at 1 µL/mL was added to cell cultures to inhibit cytokine secretion and to allow intracellular molecule detection by flow cytometry. After 16-24 h, cells were stained as described in the following.

### T-Cell Subpopulations and Intracellular IFN-γ Detection

Stimulated PBMCs were stained with fluorochrome-conjugated antibodies prepared in Brilliant Stain Buffer (BD Biosciences) (see **Supplementary Figure S1** for gating strategy**)**. The Cytofix/Cytoperm solution kit (BD Biosciences) was used for the intracellular IFN-γ staining, according to the manufacturer’s instructions (see **Supplementary Table S1** for the list of antibodies and reagents used). Dead cells were excluded from the analysis by side/forward scatter gating and then by Fixable Viability stain 700 (BD Biosciences). At least 100,000 lymphocytes from each sample were gated (except for three samples, two from the unstimulated conditions and one from the SEB condition which were gated with 80,000 events). Samples were acquired on a BD Lyric (BD Biosciences) cytometer and data were analyzed by the FlowJo software (version 10, Tree Star). IFN-γ-mediated T-cell response was considered positive when: i) the frequency of the SARS-COV-2 peptide-stimulated PBMCs was at least twofold higher compared to the unstimulated control; and ii) at least 10 events were present within the IFN-γ gate (18).

### Anti-SARS-CoV-2 Specific IgG Evaluation

The humoral response to vaccination was assessed by quantifying the anti-Nucleoprotein-IgG and the anti-RBD-IgG (Architect^®^ i2000sr Abbott Diagnostics, Chicago, IL). Anti-N-IgG were expressed as arbitrary units (AU)/mL and values were considered positive when ≥ 1.4. Anti-RBD-IgG were expressed as binding arbitrary units (BAU)/mL and values were considered positive when ≥ 7.1.

### Statistical Analysis

Data were analyzed using GraphPad (GraphPad Prism 8 XML ProjecT). Categorical variables were reported as count and proportion, whereas continuous variables, including IFN-γ levels and anti-RBD titers, were reported as median and interquartile range (IQR). Results were evaluated by non-parametric statistical inference tests. The comparisons among groups were evaluated using the Kruskal-Wallis test, whereas the Mann-Whitney U-test with Bonferroni correction was used for pairwise comparisons. The Chi-squared test was used for categorical variables. Correlations of demographic, clinical, and laboratory variables with antibody and S-specific T-cell response, as well as between-assay correlations, were assessed by non-parametric Spearman’s Rank test. Spearman’s r_ho_>0.7 was considered high correlation, 0.7 <r_ho_>0.5 moderate correlation, and r_ho_<0.5 low correlation. Two-tailed p-values were considered significant if <0.05, except for subgroup analyses by type of rheumatologic-specific treatment, where a correction for multiplicity was applied according to the Bonferroni method, yielding to a significant two-tailed p-value threshold of 0.0125 (α/4).

## Results

### Demographic and Clinical Characteristics of the Enrolled Subjects

We prospectively enrolled 202 vaccinated subjects from whom 35 were RA patients and 167 were HCWs. Significant differences were found with respect to age (p<0.0001) and origin (p=0.005), but not for sex between the two groups (**Table 1**).

The RA cohort consisted of 7 patients under treatment with TNF-α-inhibitors with or without DMARD, 8 treated with IL-6-inhibitors with or without DMARD/CCS, 13 under CTLA-4-inhibitors with or without DMARD/CCS, and 7 under only DMARD (5 patients were receiving MTX, 1 salazopyrin, and 1 hydroxychloroquine) with or without CCS. At vaccination, the median treatment duration for TNF-α-inhibitors with or without DMARD was 2.9 years (IQR: 1.3-11), for IL-6-inhibitors with or without DMARD/CCS 6.1 years (IQR: 4.9-7.6), for CTLA-4-inhibitors with or without DMARD/CCS 6 years (IQR: 1.9-10), and for DMARD with or without CCS 2.2 years (IQR: 1.9-4.9).

### Monitoring of Disease Activity at Baseline and After the Second Dose of Vaccination in RA Patients

In RA patients, the ongoing therapeutic regimen was modified during the vaccination period according to the ACR indications (19). In particular, MTX and JAK-inhibitors were stopped for one week after the first and second dose, whereas abatacept, the CTLA-4 inhibitor, was stopped one week before and after the first dose only.

No significant increase of disease activity was found at T1 compared with baseline values [T0: median 2.9, IQR (2.4-3.5) *vs* T1: median 3.1, IQR (2.0-3.5), p=0.759]. No severe adverse reactions were observed in vaccinated patients. Mild, transient, systemic, and local side effects, mainly pain at the injection location, mild fever, arthromyalgia, and fatigue, were reported by 18 patients (46%).

### Antibody-Specific Response in Vaccinated Individuals

**Figure 1:**
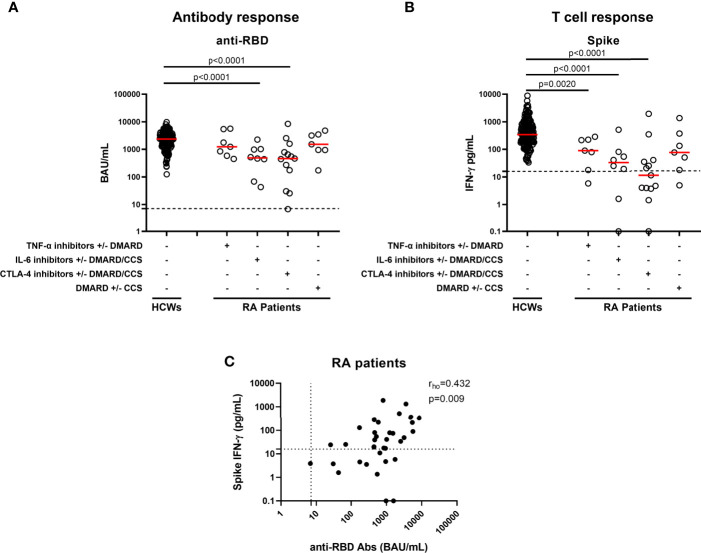
Antibody and T-cell responses elicited by SARS-CoV-2 vaccination in RA patients. Evaluation of antibody response **(A)** and IFN-γ response to spike antigen **(B)** in 167 HCWs and 35 RA patients stratified according to drug treatment in four groups: TNF-α inhibitors with or without DMARD (n=7), IL-6 inhibitors with or without DMARD/CCS (n=8), CTLA-4 inhibitors with or without DMARD/CCS (n=13), and DMARD with or without CCS (n=7). Correlation across humoral and cell-mediated immunity in RA patients **(C)** is shown. SARS-CoV-2 specific anti-RBD Abs were quantified in plasma or sera samples. Anti-RBD-IgG were expressed as binding arbitrary units (BAU)/mL and values ≥ 7.1 were considered positive. IFN-γ levels were shown as median after subtracting the background. Dashed lines identify the cut-off of each test (spike 16 pg/mL, anti-RBD 7.1 BAU/mL). Each black dot represents one sample, and the red horizontal lines represent the median. Statistical analysis was performed using the Mann-Whitney U-test with Bonferroni correction, and p ≤ 0.0125 was considered significant. Correlations between assays were assessed by non-parametric Spearman’s rank tests. A two-sided p-value <0.05 was considered statistically significant. CCS, corticosteroids; DMARD, disease modifying anti-rheumatic drugs; RA, rheumatoid arthritis; Abs, antibodies; RBD, receptor-binding-domain; HCWs, health care workers.

Humoral response was evaluated by measuring the anti-RBD antibodies, while the natural infection was excluded by the detection of anti-N-antibodies. Both HCWs and RA patients were naïve for SARS-CoV-2 infection, as confirmed by the undetectable levels of anti-N antibodies (data not shown). A detectable anti-RBD antibody response was observed in all HCWs (100%) and in all RA patients, except for one individual (97.1%). However, the magnitude of the HCWs response was significantly higher than that of RA patients under CTLA-4 and IL-6 inhibitors with or without DMARD/CCS (p<0.0001 in both groups). Differently, no significant differences were found for the anti-RBD antibody response of patients under TNF-α-inhibitors with or without DMARD (p=0.273) and DMARD with or without CCS (p=0.421) (**Figure 1A**). The response to vaccination can naturally wane with age. Older age may have an impact on the magnitude of the humoral response (19). Therefore, among the HCWs we selected a group (n=50) who were age-matched [age median: 56, IQR (53–61)] with the RA cohort. We confirmed the results described in **Figure 1A** and we suggest that, more than the age, the RA related-therapies are likely responsible for the reduced specific-antibody response (**Supplementary Figure S2A**).

### SARS-CoV-2-S-Specific T-Cell Response in Vaccinated Individuals

All HCWs showed an IFN-γ-S-specific T-cell response, evaluated by the whole blood platform (3, 10). Contrarily, significant different proportions of responders were found in RA patients under both CTLA-4 and IL-6-inhibitors with or without DMARD/CCS therapy, compared to HCWs (p=0.0018 and p<0.0001, respectively) (**Table 2**). Moreover, the quantitative responses were significantly different among groups (p<0.0001) (**Figure 1B**). In particular, the IFN-γ-S-specific levels were significantly lower in RA patients under TNF-α-inhibitors with or without DMARD, IL-6-inhibitors with or without DMARD/CCS, and CTLA-4-inhibitors with or without DMARD/CCS therapy than those in HCWs (p=0.0020, p<0.0001, p<0.0001, respectively). In contrast, no significant difference was found between the IFN-γ-S-specific response of patients treated with DMARD with or without CCS compared to that of HCWs (p=0.016), albeit the IFN-γ levels were lower than those of HCWs. These data were confirmed comparing the S-specific T-cell response of RA patients with that of age-matched HCWs (n=50) [age median: 56, IQR (53–61)] (**Supplementary Figure S2B**).

**Table 2:** Serological and T-cell specific response.

	Characteristics			RA patients	HCWs	P value	
			**N (%)**	35 (17.3)	167 (82.7)		
**Antibody response**	**Qualitative response**	**Anti-RBD abs responders** **N (%)**		34 (97)	167 (100)		0.028^§^
		**Anti-RBD abs responders within the subgroups** **N (%)**	**TNF-α-inhibitors +/- DMARD**	7/7 (100)	–	0.627^§^	>0.999^§^
			**IL-6- inhibitors+/-DMARD/CCS**	8/8 (100)	–		>0.999^§^
			**CTLA-4- inhibitors +/-DMARD/CCS**	12/13 (92.3)	–		0.072^§^
			**DMARD +/- CCS**	7/7 (100)	–		>0.999^§^
	**Quantitative response**	**Anti-RBD abs** **BAU/mL Median (IQR)**		784.7 (441–1763)	2351 (1389–3748)		<0.0001*
			**TNF-α-inhibitors +/- DMARD**	1239 (589–5426)	–	<0.0001^#^	0.273*
			**IL-6-inhibitors+/-DMARD/CCS**	492 (161–1007)	–		<0.0001*
			**CTLA-4-inhibitors +/-DMARD/CCS**	465 (103–1189)	–		<0.0001*
			**DMARD +/- CCS**	1526 (943–3471)	–		0.421*
**Spike specific IFN-γ T cell response**	**Qualitative response**	**Anti-S responders N (%)**		24 (69)	167 (100)		<0.0001^§^
		**Anti-S responders within the subgroups** **N (%)**	**TNF-α-inhibitors +/- DMARD**	6/7 (86)	–	0.165^§^	0.040^§^
			**IL-6-inhibitors+/-DMARD/CCS**	6/8 (75)	–		0.0018^§^
			**CTLA-4-inh-bitors +/-DMARD/CCS**	6/13 (46)	–		<0.0001^§^
			**DMARD +/- CCS**	6/7 (86)	–		0.040^§^
	**Quantitative response**	**Anti-S IFN-γ** **pg/mL Median (IQR)**		34 (4.7-130)	343 (188–756)		<0.0001*
			**TNF-α-inhibitors +/- DMARD**	89.6 (17.8-224)	–	<0.0001^#^	0.0020*
			**IL-6-inhibitors+/-DMARD/CCS**	33.2 (6.1-73.9)	–		<0.0001*
			**CTLA-4-inhibitors +/-DMARD/CCS**	10.9 (3.7-36.7)	–		<0.0001*
			**DMARD +/-CCS**	74.6 (17.2-364)	–		0.016*

DMARD, disease modifying antirheumatic drugs; CCS, corticosteroids; RA, rheumatoid arthritis; N, number; IQR, interquartile range; abs, antibodies; RBD, receptor-binding-domain; S, spike; ^§^Chi-square test; *Mann-Whitney U-statistic test; ^#^Kruskal-Wallis test.

### Correlation Between Anti-RBD Antibody Titer, S-Specific T-Cell Response, and Lymphocyte Number

We then focused on the correlation between the two arms of the immune response. A significant correlation between anti-RBD-antibody titer and SARS-CoV-2-S-specific IFN-γ T-cell response was found in HCWs (3). Similarly, a significant moderate correlation was observed in RA patients (rho=0.432, p=0.009) (**Figure 1C**). Differently, there was no correlation between the lymphocyte number and anti-RBD antibody titer (rho=0.325, p=0.069) or T-specific response (rho=0.151, p=0.409) (data not shown). Further, we analyzed in the RA cohort the impact of age, gender, or years of therapy to identify potential factors affecting the qualitative and quantitative immune responses. None of these variables showed a significant impact on the humoral- or T-cell-specific responses (**Supplementary Tables S2, S3**).

### IFN-γ Response to Spike Is Mainly Mediated by CD4^+^ T Cells

**Figure 2:**
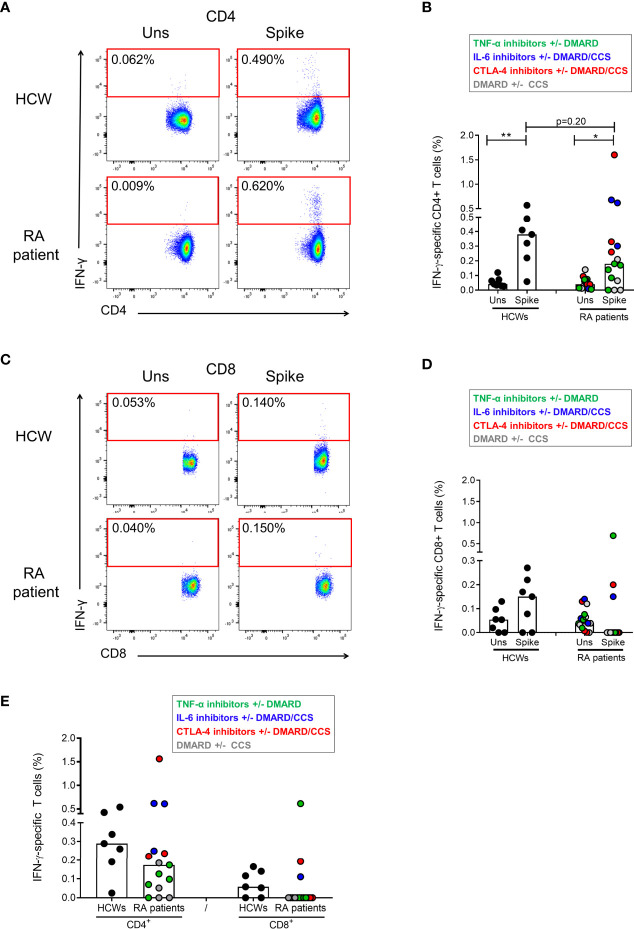
Characterization of the IFN-γ-S-specific T-cell response by flow cytometry. PBMCs from HCWs (n=7) and RA patients (n=15) were *in vitro* stimulated for 24 h with spike peptide pool and the frequency of IFN-γ-specific T cells was evaluated by flow cytometry. **(A)** Plots show the frequency of IFN-γ in a representative HCW subject and RA patient within the CD4^+^ T-cell subpopulations. **(B)** CD4^+^ T-cell-specific response compared to the unstimulated condition is shown in HCWs and RA patients. **(C)** Plots show the frequency of IFN-γ in a representative HCW subject and RA patient within the CD8^+^ T-cell subpopulations. **(D)** CD8^+^ T-cell-specific response compared to the unstimulated condition is shown in HCWs and RA patients. **(E)** Frequency of the CD4^+^ and CD8^+^ T-cell response (after subtraction of the unstimulated-condition value) is shown in HCWs and RA patients. Each dot represents a different HCW or RA individual and black lines represent the median. RA patients were color-coded, and each color corresponds to a different administered treatment, as shown in the figure legend. Statistical analysis was performed using the Mann-Whitney test and p-value was considered significant if ≤0.05. *p = 0.012 and **p = 0.004. IFN, interferon; RA, rheumatoid arthritis; HCWs, health care workers.

To assess which T-cell subset among CD4^+^ or CD8^+^ T cells was responsible for the SARS-CoV-2-S-specific response, the IFN-γ-S-specific T-cell frequency was analyzed by flow cytometry after stimulation of the PBMCs with the spike peptide pool. To this aim, we used PBMCs isolated from 7 HCWs and 15 RA patients. Among the RA subjects, we selected those characterized by good specific antibody and T-cell responses (**Figure 2**). In particular, we selected 5 RA patients under TNF-α-inhibitors with or without DMARD, 3 patients under CTLA-4-inhibitors with or without DMARD/CCS, 3 patients under IL-6-inhibitors with or without DMARD/CCS, and 4 DMARD with or without CCS-treated patients. We show that the IFN-γ response is mediated by CD4^+^ T cells following *in vitro* stimulation with spike peptide pool compared to the unstimulated control, both in HCWs and in RA patients (**Figures 2A, B**). The CD4^+^ T-cell response was scored positive in all HCWs (7/7, 100%) (**Figure 2E**) and in most RA patients (12/15, 80%) (**Figure 2E**). The IFN-γ response was mediated also by CD8^+^ T cells in most HCWs (5/7, 71%) and in a portion of the RA patients tested (3/15, 20%) (**Figures 2C** **, D**). In the HCWs, the magnitude of the specific response was higher in CD4^+^ T cells (median: 0.28%, IQR: 0.19-0.42) (**Figure 2E**) compared to CD8^+^ T cells (median: 0.058%, IQR: 0.00-0.14) (**Figure 2E**). Similarly, in RA patients the CD4^+^ T cells showed a higher specific response (median: 0.17%, IQR: 0.05-0.24) (**Figure 2E**) compared to the CD8^+^ T cells (median: 0.00%, IQR: 0.00-0.00) (**Figure 2E**). All HCWs and RA patients responded to SEB, used as a positive control, confirming the absence of an impairment of the cytokine production (**Supplementary Figures S3A, B**). Interestingly, in the RA cohort, the CD8^+^ T-cell mediated response to SEB was significantly higher compared to that observed in HCWs (p=0.001) (**Supplementary Figure S3B**).

## Discussion

Mass vaccination is a crucial public health measure for limiting COVID-19 pandemic especially in fragile populations such as RA patients. Here, we show the results of the immune response to BNT162b2 vaccine in RA patients that were vaccinated based on the ACR indications (20) with an interruption of MTX and JAK-inhibitors 1 week after the first and second vaccine dose, or with an interruption of abatacept for 1 week before and after the first dose.

In RA patients, BNT162b2 vaccine showed a good safety profile and disease activity remained stable with no patient experiencing a disease relapse. The vaccine induced an antibody-specific response in almost all patients (97%), although the titer was significantly reduced in those under CTLA-4-inhibitors (abatacept) or IL-6-inhibitors compared to HCWs. Concomitantly, spike-specific T-cell response was evaluated and scored positive in 69% of RA patients vs. 100% of HCWs with significantly lower levels in those under a biological therapy compared to HCWs, particularly in patients under CTLA-4-inhibitors or IL-6-inhibitors. The response to vaccination may decrease with age. However, the present finding was confirmed when comparing the results of a group of age-matched HCWs with the RA cohort suggesting that the lower magnitude of both RBD-antibody and T-cell responses was not due to the older age of patients but likely to the RA-treatment. Based on these results, we confirm that COVID-19 vaccination is immunogenic and safe, also in patients under immunosuppressive therapies, although the specific immune responses were present at a lower magnitude compared to the healthy population.

Recently, it has been shown that individuals with immune-mediated inflammatory diseases treated with MTX have up to a 62% rate of specific-immune response to BNT162b2 mRNA vaccine (14), whereas those under cytokine-inhibitors have levels similar to those of healthy controls (greater than 90%) (11, 13, 14). The different results reported here on the impact of immunosuppressant drugs on the humoral response may be associated with the therapeutic strategy adopted to optimize vaccine immunogenicity. Indeed, as already seen for seasonal influenza vaccination (21), the 1-week interruption of MTX after the first and second vaccine dose may have reduced the negative impact on antibody production previously shown (11, 14). This strategy was useful also for those under CTLA-4-inhibitors, as demonstrated by the reassuring proportion of patients mounting an anti-RBD-specific response rate, 92% here *vs*. 62% of previous reports (11, 14), although with a significant decreased antibody titer compared to that observed in the HCWs. Furthermore, notably, this brief “window of therapy interruption” did not affect the RA disease activity, as shown by the DAS28crp that remained stable throughout the vaccination period.

On the other hand, the strategy of interrupting abatacept administration limited to the first dose was not satisfactory to provide the induction of the T-cell-specific response, which is known to be impaired by the drug itself (22), as shown here by the 46% positive responder rate. Based on these results and considering that abatacept blocks the T-cell activation by binding with high-affinity CD80/CD86 molecules thus interfering with the co-stimulation signals delivered through the antigen presenting cells (23), it may be reasonable to extend its interruption also at the second vaccine dose. This approach may improve the induction of a specific immune response especially at the T-cell level.

Interestingly, IL-6-inhibitors had a higher negative impact on the magnitude of antibody- and T-cell-specific Responses compared to TNF-α-inhibitors. This is likely due to the important effect of IL-6 in controlling the survival, population expansion, and maturation of B cells and plasmablasts acting on the follicular helper T cells, a specialized subset of CD4^+^ T cells that localize to B cell follicles, where they promote B cell proliferation and immunoglobulin class switching (24, 25). IL-6 is also important for the T-cell memory response (24, 26).

Flow cytometry analysis showed that the *in vitro* T-cell response to SARS-CoV-2 spike peptides is mediated by CD4^+^ and CD8^+^ T cells in both HCWs and RA patients. Notably, CD4^+^ T-cell frequency was higher compared to that observed for the CD8^+^ T cells. These data agree with the results from the HCWs cohorts (5, 27–31) and the COVID-19 convalescent subjects (17, 32). Remarkably, CD8^+^ T-cell response was lower in the RA cohort compared to that observed in the HCWs. Due to the small sample size of samples included in the flow cytometry analysis, we cannot associate the low frequency of the CD8^+^ T cell response observed in RA patients to the different therapeutic regimens.

Some limitations of this study need to be considered. First, it was a single center study with a low number of recruited patients that may limit the impact of the study, especially for the comparison of the effects of vaccination among different immunosuppressive therapies. However, the enrolled patients are representative of RA patients under different therapies, and they were well characterized, both clinically and immunologically. Second, the evaluation of the immune responses was performed at a single time point post-vaccination, and the assay used to detect the T-cell response was based on the measurement of a single cytokine (IFN-γ) differently from published studies assessing additional T-helper 1 cytokines (4). However, it was shown that the IFN-γ-specific T-cell response correlates with RBD-antibody titers (4); therefore this cytokine may be considered as a robust parameter to detect T-cell-specific response induced after vaccination.

Importantly, one of the strengths of this study is the more accurate assessment of the humoral immune response using specific anti-RBD-IgG against the total spike protein compared to the previous published work, where IgG antibody titers against only S1 were evaluated (14). In addition, we characterized the T-cell response in terms of CD4^+^ or CD8^+^ T-cell involvement. The assays used in the present study to detect SARS-CoV-2 specific response are easy and highly reproducible (17, 33–38), and therefore are compatible with the routine monitoring of vaccinated individuals (4, 5, 16). Indeed, the T-cell response was detected by a whole blood assay, whose platform is similar to current tests measuring T-cell-specific responses against *M*. *tuberculosis* in both immune-competent and immune-suppressed subjects (39–42).

To the best of our knowledge, this is the first study evaluating both humoral and cellular immune responses to BNT162b2 vaccine in RA patients, who underwent a temporary suspension of immunosuppressive treatment during vaccine administration. For the optimal management of RA patients, clinicians need to consider both the risk of disease relapse and that of a decreased vaccine immunogenicity. These findings suggest that holding treatment with MTX and abatacept at the first and second vaccine dose can be considered a useful practice in clinically stable patients. To draw definite conclusions, these results need to be confirmed in a larger population adopting a similar therapeutic strategy suspension, and future studies are needed to further evaluate the longevity of humoral and T-cell responses following vaccination.

## Data Availability Statement

The raw data generated and/or analyzed within the present study are available in our institutional repository (rawdata.inmi.it), subject to registration. The data can be found by selecting the article of interest from a list of articles ordered by year of publication. No charge for granting access to data is required. In the event of a malfunction of the application, the request can be sent directly by e-mail to the Library (biblioteca@inmi.it).

## Ethics Statement

The studies involving human participants were reviewed and approved by the Ethical Committee of the National Institute of Infectious Diseases “Lazzaro Spallanzani” IRCCS (approval numbers 297/2021 and 318/2021). The patients/participants provided their written informed consent to participate in this study.

## Author Contributions

DG and EN wrote the project to be submitted to the Ethical Committtee. DG, EN, AP, BL, CA, and CC conceived and designed the study. Experiments were performed by AA, SM, DL, EC, GG, VV, AS, FR, AMGA, SNF, and CF performed the flow cytometry analysis. AA and SL performed the statistical analysis. AP, BL, GC, RR, SS, GN, GM, CP, and SV enrolled patients and collected clinical data. AP, AA, DG, BL, VP, MC, GI, and EN drafted the article or revised it critically. All authors contributed to the article and approved the submitted version.

## Funding

This work was supported by INMI “Lazzaro Spallanzani” Ricerca Finalizzata COVID-2020-12371675 and Ricerca Corrente on emerging infections both funded by Italian Ministry of Health, and by generous liberal donations funding for COVID-19 research from Esselunga S.p.A, Camera di Commercio, Industria e Artigianato di Roma, Società Numero Blu Servizi S.p.A., Fineco Bank S.p.A, Associazione magistrati della Corte dei conti, and Società Mocerino Frutta Secca s.r.l (resolutions n°395 of May 25^th^ 2021, n°254 of April 24^th^ 2021 and n°257 of April 14^th^ 2021). The funders were not involved in the study design, collection, analysis, and interpretation of data, the writing of this article, or the decision to submit it for publication.

## Conflict of Interest

EN is a member of the advisory board by Gilead, Lilly and Roche and received fees for educational training by Gilead, Lilly and Roche. DG is member of the advisory board by Biomerieux and Eli-Lilly and received fees for educational training or consultancy by Biogen, Cellgene, Diasorin, Janssen, Qiagen, and Quidel.

The remaining authors declare that the research was conducted in the absence of any commercial or financial relationships that could be construed as a potential conflict of interest.

## Publisher’s Note

All claims expressed in this article are solely those of the authors and do not necessarily represent those of their affiliated organizations, or those of the publisher, the editors and the reviewers. Any product that may be evaluated in this article, or claim that may be made by its manufacturer, is not guaranteed or endorsed by the publisher.
