# Supplementary material for: ImmunosuppressiveTherapies Differently Modulate Humoral- and T-Cell-Specific Responses to COVID-19 mRNA Vaccine in Rheumatoid Arthritis Patients
Source: Front Immunol. 2021 Sep 14;12:740249. doi: 10.3389/fimmu.2021.740249 (PMC8477040; doi:10.3389/fimmu.2021.740249)
Supplement: Supplementary file 1 [file DataSheet_1.docx]

Supplementary Material

1. **Supplementary Tables**

**Supplementary Table S1**

**Table S1.** List of antibodies and reagents for flow cytometry analysis

| **Antibodies/Reagents** | **Clone and Cat#** | **From** | |
| --- | --- | --- | --- |
| CD3 PE-Cy7  CD3 PE | Clone SK7; Cat# 341111  Clone SP34-2; Cat#552127 | BD | |
| CD4 BV711 | Clone SK3; Cat# 63028 | BD | |
| CD8 APC-H7 | Clone SK1; Cat# 41400 | BD | |
| IFNγ BV421 | Clone B27; Cat# 562988 | BD | |
| Fixable Viability stain 700  APC-R700 | Cat#564997 | BD | |
| Brilliant stain Buffer | 563794 | BD |  |
| Cytofix/Cytoperm solution kit | 554714 | BD |  |

**Supplementary Table S2**

**Table S2. Association between demographic and clinical characteristics of RA patients and anti-S/RBD response**

|  | **Adjusted multivariable RR** | |
| --- | --- | --- |
|  | **RR (95%CI)** | **P value** |
| **Gender:** |  |  |
| **Female vs Male** | 1.26 (0.33-4.85) | 0.7319 |
| **Age group:** |  |  |
| **>= 60 vs 39-59** | 0.95 (0.28-3.21) | 0.9336 |
| **Years of therapy:** |  |  |
| **>=5 vs <=5** | 0.53 (0.15-1.85) | 0.3074 |
| **Treatment:** |  |  |
| **IL-6-inhibitors +/-DMARD/Cortisone vs TNF-α-inhibitors +/- DMARD** | 0.30 (0.05-1.67) | 0.1608 |
| **CTLA-4-inhibitors +/-DMARD/Cortisone vs TNF-α-inhibitors +/- DMARD** | 0.25 0.05 -1.21) | 0.0827 |
| **DMARD +/- Cortisone vs TNF-α-inhibitors +/- DMARD** | 0.79 (0.13-4.81) | 0.7936 |

**Supplementary Table S3**

**Table S3. Association between demographic and clinical characteristics of RA patients and Spike-IFN-γ-specific T-cell response**

|  | **Adjusted multivariable RR** | |
| --- | --- | --- |
|  | **RR (95%CI)** | **P value** |
| **Gender:** |  |  |
| **Female vs Male** | 1.88 (0.26-13.73) | 0.52234 |
| **Age group:** |  |  |
| **>= 60 vs 39-59** | 1.05 (0.18-6.32) | 0.95296 |
| **Years of therapy:** |  |  |
| **>=5 vs <=5** | 1.83 (0.29-11.54) | 0.50834 |
| **Treatment:** |  |  |
| **IL-6-inhibitors +/-DMARD/Cortisone vs TNF-α-inhibitors +/- DMARD** | 0.19 (0.01-2.47) | 0.19691 |
| **CTLA-4-inhibitors +/-DMARD/Cortisone vs TNF-α-inhibitors +/- DMARD** | 0.15 (0.01-1.58) | 0.11029 |
| **DMARD +/- Cortisone vs TNF-α-inhibitors +/- DMARD** | 1.18 (0.08-16.82) | 0.90078 |

1. **Supplementary Figures**

**Supplementary Figure S1**


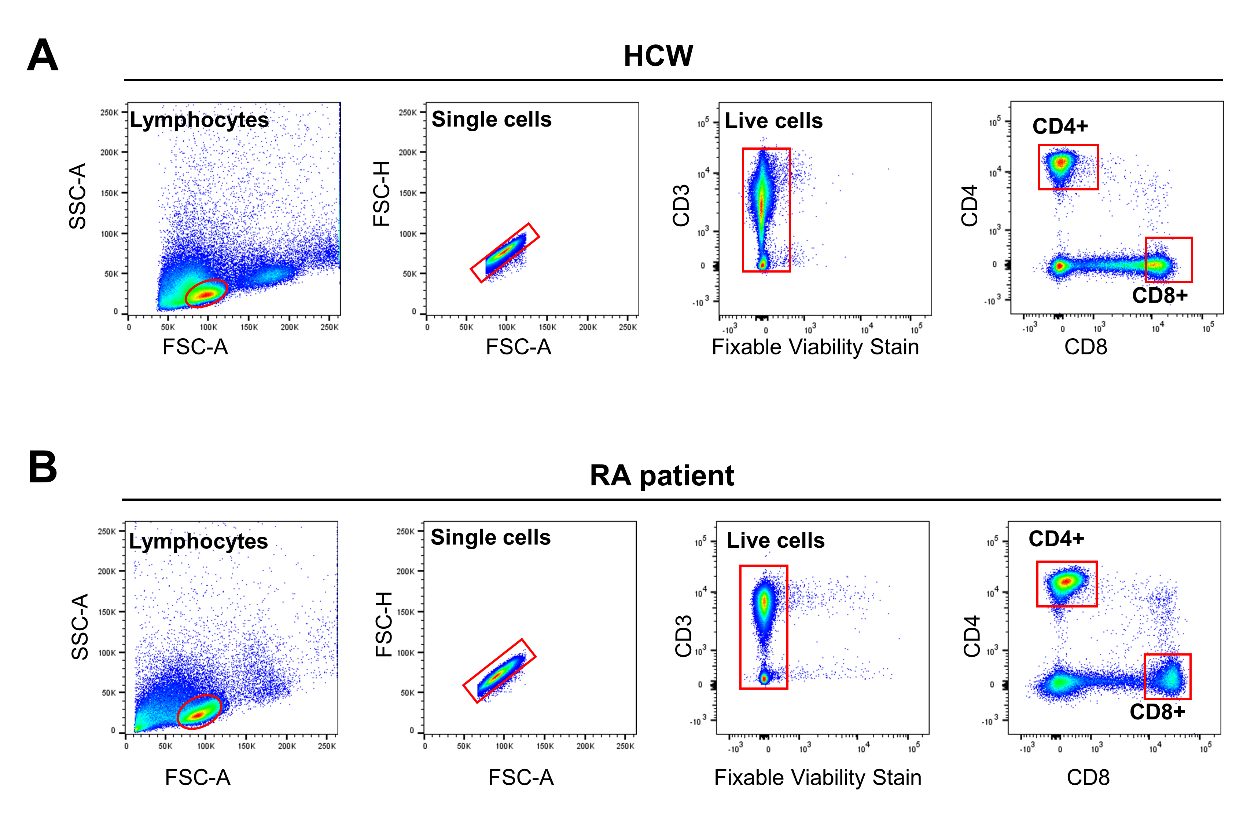


**Supplementary Figure S1. Flow cytometry gating strategy.** T-cell subpopulations were gated as described in a representative HCW individual (**A**) and one RA patient (**B**) according to the expression of surface markers.

**Supplementary Figure S2**

**
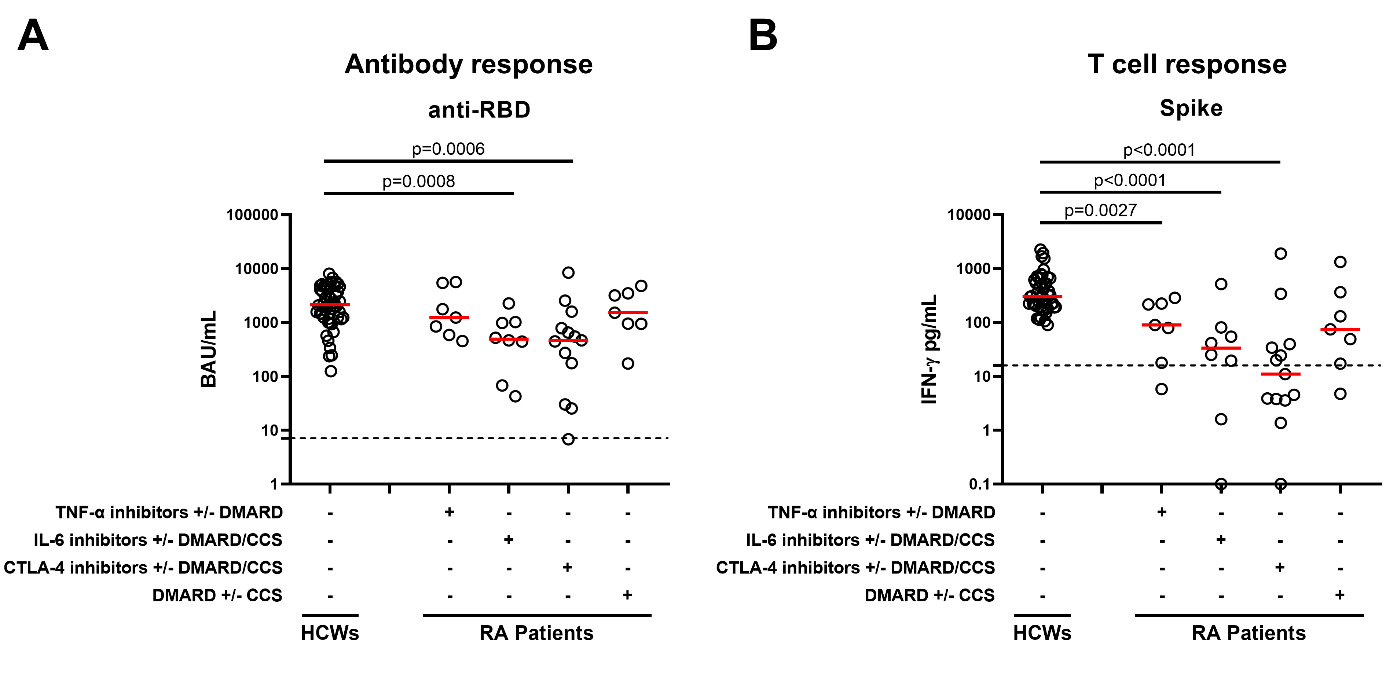
**

**Supplementary Figure S2. Antibody and T-cell responses induced by SARS-CoV-2 vaccination in rheumatoid arthritis patients.** Characterization of antibody response (**A**) and IFN-γ response to spike peptides (**B**) in 50 HCWs who were age-matched [age median 56, IQR (53-61)] with 35 RA patients [age median 59, IQR (55-65)] stratified according to the drug treatment in four groups: TNF-α inhibitors with or w/o DMARD (n=7), IL-6 inhibitors with or w/o DMARD/CCS (n=8), CTLA-4 inhibitors with or w/o DMARD/CCS (n=13) and DMARD with or w/o CCS (n=7). SARS-CoV-2 specific anti-RBD Abs were quantified in plasma or sera samples. Anti-RBD-IgG values were expressed as Binding Arbitrary Units (BAU)/mL and values ≥ 7.1 were considered positive.

Medians of IFN-γ are shown (horizontal red lines) and are calculated based on the values obtained in the S-stimulated samples subtracted from the unstimulated control value. Each black dot represents one sample. Dashed lines identify the cut-off of each test (spike 16 pg/mL, anti-RBD 7.1 BAU/mL). Statistical analysis was performed using the Mann-Whitney U-test with Bonferroni correction, and p≤0.0125 was considered significant. **Footnotes:** CCS, Corticosteroids; DMARD, Disease Modifying Anti-Rheumatic Drugs; RA, Rheumatoid arthritis; Abs, antibodies; RBD, Receptor-Binding-Domain; HCWs, Health Care Workers.

**Supplementary Figure S3**

**
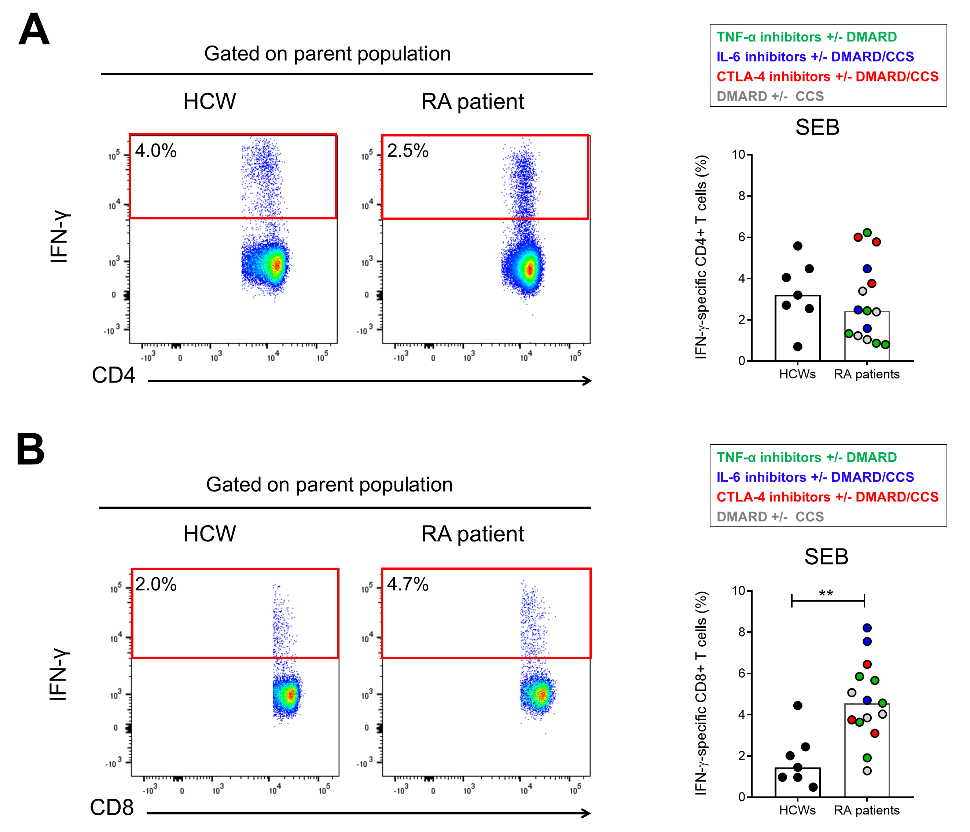
**

**Supplementary Figure S3. CD4^+^ and CD8^+^ T-cell response to SEB.** PBMCs from HCWs (n=7) and RA patients (n=15) were *in vitro* stimulated for 24h with SEB, as positive control. IFN-γ response to SEB stimulus was detected in both HCWs and RA individuals in CD4^+^ T cells **(A)** and CD8^+^ T cells **(B)**. Each dot represents a different HCW or RA individual. RA patients were indicated with different colors based on the pharmacological treatment, as described in the figure legend. Black lines represent the median. Statistical analysis was performed using the Mann-Whitney test and p value was considered significant if ≤0.05. ** p=0.003. **Footnotes:** IFN, Interferon; RA, rheumatoid arthritis; HCWs, Health Care Workers; SEB, Staphylococcal Enterotoxin B.
